# Supplementary material for: The relationships between impulsivity and mood in bipolar disorder: An ecological momentary assessment study
Source: PLoS One. 2025 Jul 2;20(7):e0314963. doi: 10.1371/journal.pone.0314963 (PMC12221055; doi:10.1371/journal.pone.0314963)
Supplement: S1 Appendix — (DOCX) [file pone.0314963.s001.docx]

****S1 Appendix.** Diagnostics and Assumption Checks for the logistic mixed models.**

**Model 1. Model Diagnostics and Assumption Checks. Concurrent model with mood as dependent variable**

**1. Residual Diagnostics – DHARMa Package**

**a. Dispersion Test**
DHARMa nonparametric dispersion test via sd of residuals fitted vs. simulated
Data: simulationOutput
Dispersion = 0.955
p-value = 0.704
Alternative hypothesis: two.sided

**b. Zero-Inflation Test**
DHARMa zero-inflation test via comparison to expected zeros with simulation under H0
Data: simulationOutput
Ratio observed/expected zeros = 0.950
p-value = 0.664
Alternative hypothesis: two.sided

Interpretation: Neither overdispersion nor zero-inflation was detected in the model residuals.

**Supplementary Figure 1. Histogram of Expected vs. Simulated Zeros (DHARMa Zero-Inflation Test)**


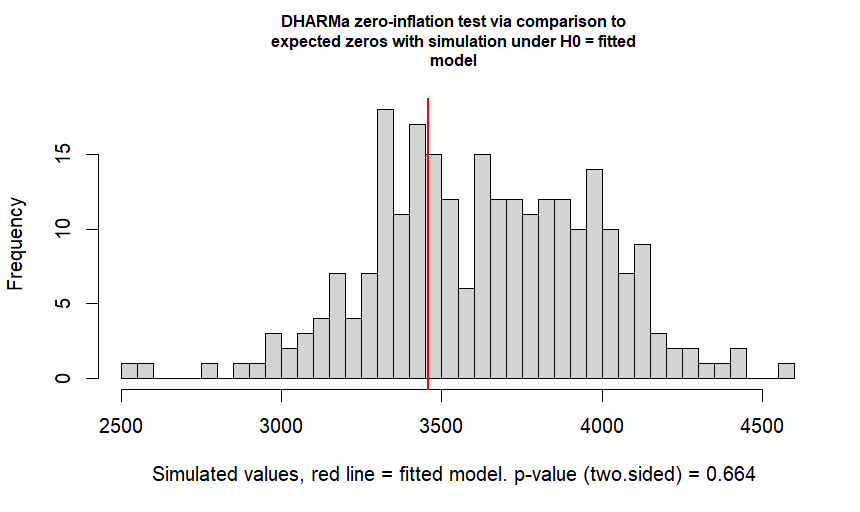
Interpretation: The distribution of simulated zeros matches the observed frequency, indicating no evidence of zero-inflation.

**2. Residuals vs. Predictor**

Plot of standardized residuals against ImpulTotal_Center (rank-transformed) to assess non-linearity or heteroscedasticity.

**Supplementary Figure 2. Residuals vs. Impulsivity Predictor (DHARMa)**


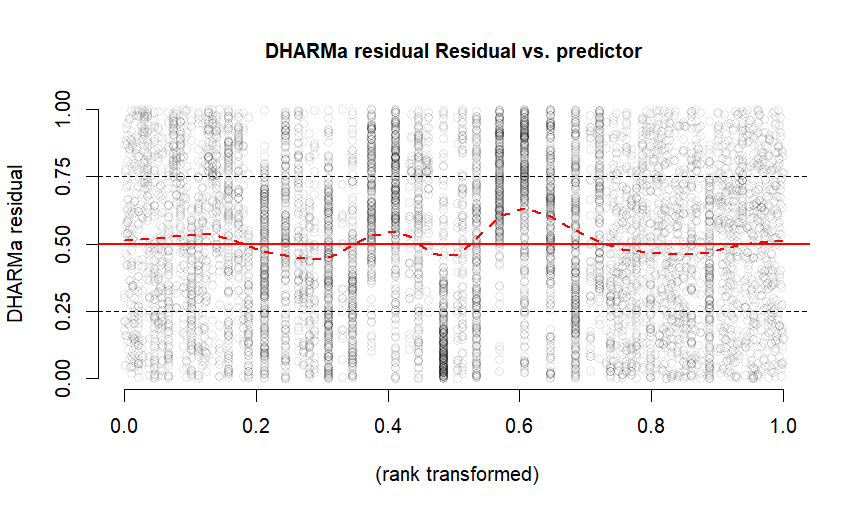
Interpretation: No clear pattern is visible in the residuals, suggesting the absence of major violations in linearity or homoscedasticity with respect to impulsivity.

**Supplementary Figure 3. QQ-plot of Random Effects**


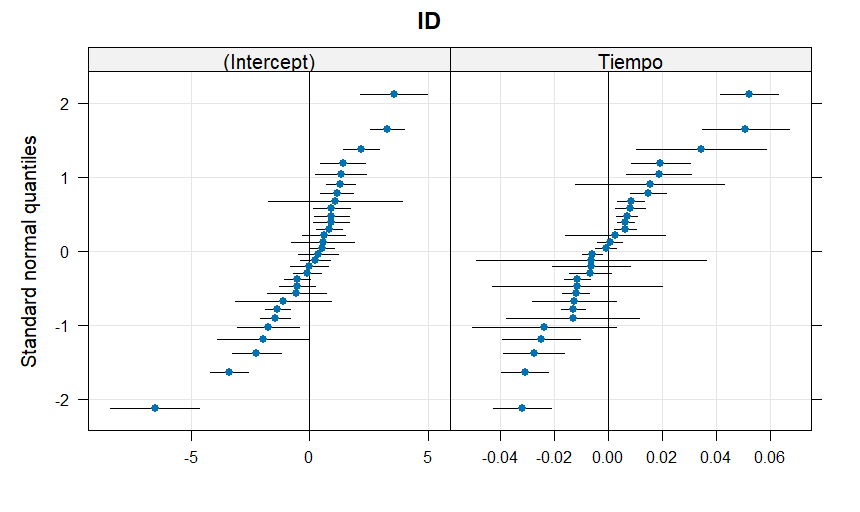
Interpretation: Both the intercept and slope random effects closely follow the expected normal distribution, supporting the assumption of normality in the random effects structure.

**4. Multicollinearity Diagnostics – VIF (Linear Approximation)**

Variance Inflation Factors (VIF) from an approximate linear model (lm):

- Time: 1.037
- Impulsivity (Time invarying):: 1.475
- Impulsivity (Time varying):: 1.010
- Diagnosis: 1.199
- Age: 1.239
- Gender : 1.184
- Family: 1.173

Interpretation: All VIF values are well below 5, indicating no problematic collinearity.

**5. Model Fit – Pseudo-R² for Generalized Mixed Model (MuMIn package)**

Function used: r.squaredGLMM(mod_granMaxfun)

- Marginal R² (fixed effects only): 0.258
- Conditional R² (fixed + random effects): 0.804

_Interpretation: Fixed effects explain ~26% of the variance; ; the full model including random effects explains ~80%.

**Model 2. Model Diagnostics and Assumption Checks. Lagged model with Mood as dependent variable**

**1. Residual Diagnostics – DHARMa Package**

**a. Dispersion Test**
DHARMa nonparametric dispersion test via sd of residuals fitted vs. simulated
Data: simulationOutput
Dispersion = 0.966
p-value = 0.760
Alternative hypothesis: two.sided

**b. Zero-Inflation Test**
DHARMa zero-inflation test via comparison to expected zeros with simulation under H0
Data: simulationOutput
Ratio observed/expected zeros = 0.946
p-value = 0.624
Alternative hypothesis: two.sided

Interpretation: Neither overdispersion nor zero-inflation was detected in the model residuals.

**Supplementary Figure 4. Histogram of Expected vs. Simulated Zeros (DHARMa Zero-Inflation Test)**


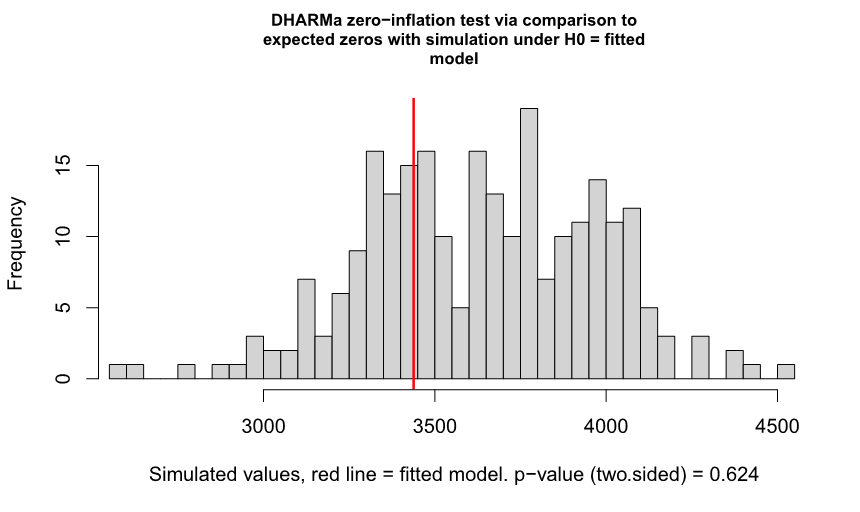


Interpretation: The distribution of simulated zeros matches the observed frequency, indicating no evidence of zero-inflation.

**2. Residuals vs. Predictor**

Plot of standardized residuals against ImpulTotal_Center (rank-transformed) to assess non-linearity or heteroscedasticity.

**Supplementary Figure 5. Residuals vs. Impulsivity Predictor (DHARMa)**


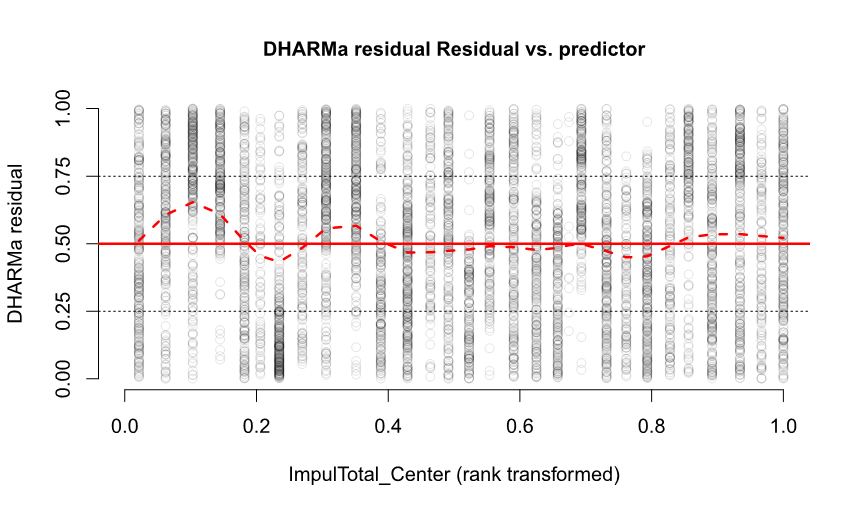
Interpretation: No clear pattern is visible in the residuals, suggesting the absence of major violations in linearity or homoscedasticity with respect to impulsivity.

**3. Normality of Random Effects**

QQ-plots of random intercepts and slopes to visually inspect normality assumptions.

**Sup**
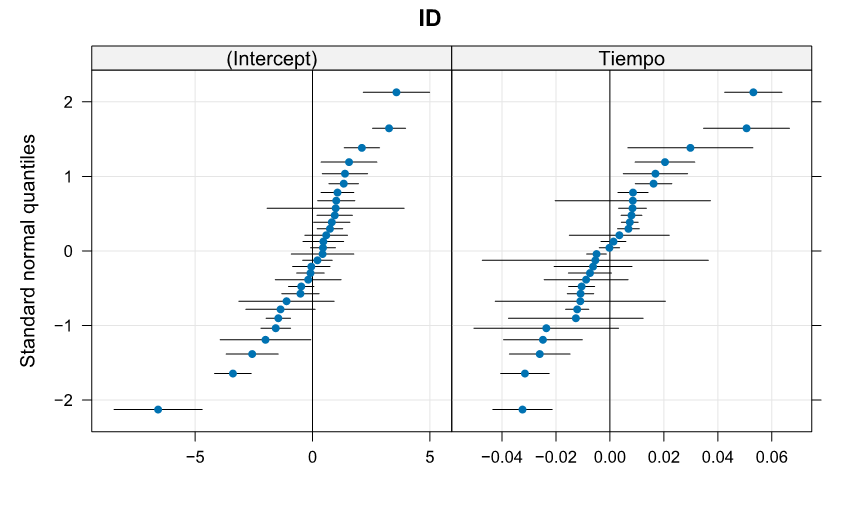
**plementary Figure 6. QQ-plot of Random Effects**

Interpretation: Both the intercept and slope random effects closely follow the expected normal distribution, supporting the assumption of normality in the random effects structure.

**4. Multicollinearity Diagnostics – VIF (Linear Approximation)**

Variance Inflation Factors (VIF) from an approximate linear model (lm):

- Time: 1.041
- Impulsivity (Time invarying): 1.301
- Impulsivity (Time varying): 1.003
- Diagnosis: 1.304
- Age: 1.131
- Gender : 1.259
- Family: 1.029

Interpretation: All VIF values are well below 5, indicating no problematic collinearity.

**5. Model Fit – Pseudo-R² for Generalized Mixed Model (MuMIn package)**

Function used: r.squaredGLMM(mod_granMaxfun)

- Marginal R² (fixed effects only): 0.267
- Conditional R² (fixed + random effects): 0.808

_Interpretation: Fixed effects explain ~27% of the variance; ; the full model including random effects explains ~81%.

**Model 3. Model Diagnostics and Assumption Checks. Concurrent model with impulsivity as dependent variable**

**1. Residual Diagnostics – DHARMa Package**

**a. Dispersion Test**
DHARMa nonparametric dispersion test via sd of residuals fitted vs. simulated
Data: simulationOutput
Dispersion = 0.927
p-value = 0.560
Alternative hypothesis: two.sided

**b. Zero-Inflation Test**
DHARMa zero-inflation test via comparison to expected zeros with simulation under H0
Data: simulationOutput
Ratio observed/expected zeros = 1.015
p-value = 0.912
Alternative hypothesis: two.sided

Interpretation: Neither overdispersion nor zero-inflation was detected in the model residuals.

**Supplementary Figure 7. Histogram of Expected vs. Simulated Zeros (DHARMa Zero-Inflation Test)**


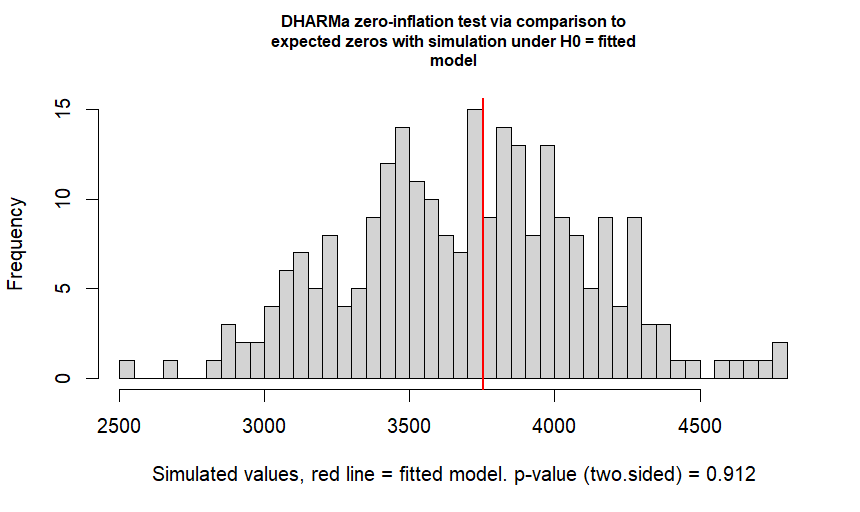


Interpretation: The distribution of simulated zeros matches the observed frequency, indicating no evidence of zero-inflation.

**2. Residuals vs. Predictor**

Plot of standardized residuals against ImpulTotal_Center (rank-transformed) to assess non-linearity or heteroscedasticity.

**Supplementary Figure 8. Residuals vs. Impulsivity Predictor (DHARMa**


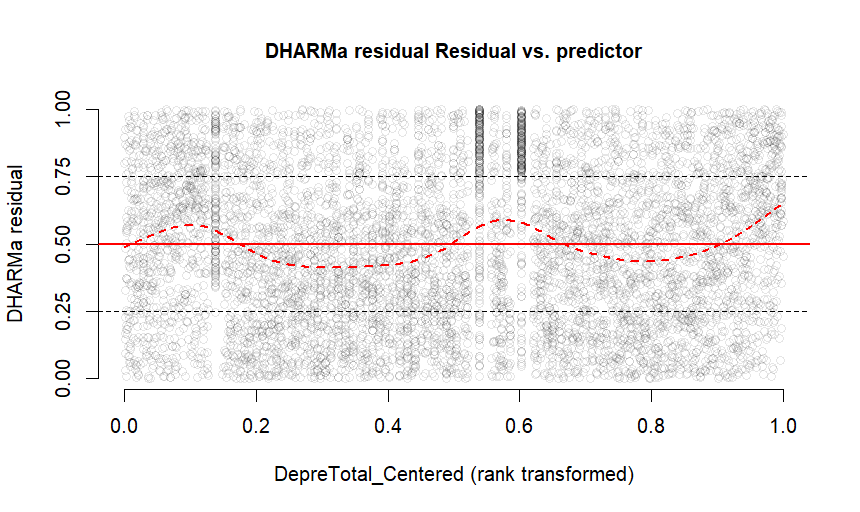
Interpretation: No clear pattern is visible in the residuals, suggesting the absence of major violations in linearity or homoscedasticity with respect to impulsivity.

**3. Normality of Random Effects**

QQ-plots of random intercepts and slopes to visually inspect normality assumptions.

**Supplementary Figure 9. QQ-plot of Random Effects**


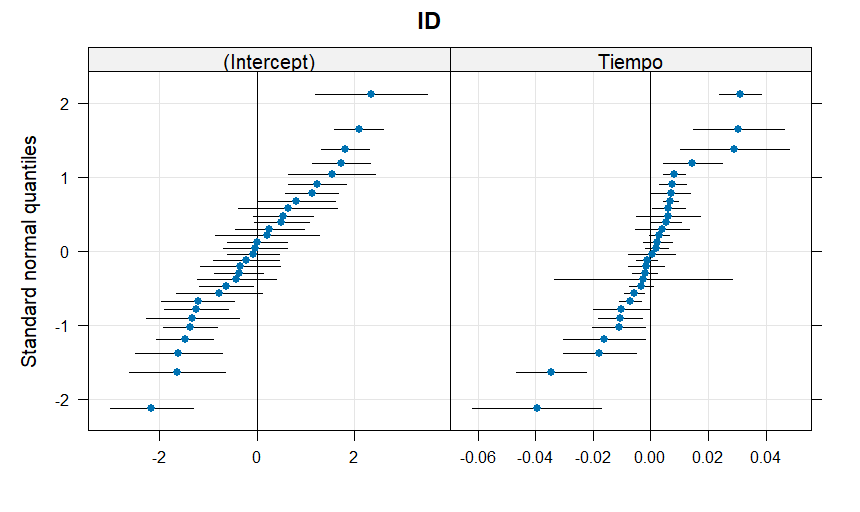
Interpretation: Both the intercept and slope random effects closely follow the expected normal distribution, supporting the assumption of normality in the random effects structure.

**4. Multicollinearity Diagnostics – VIF (Linear Approximation)**

Variance Inflation Factors (VIF) from an approximate linear model (lm):

- Time: 1.049
- Depression (Time invarying): 1.545
- Depression (Time varying): 1.001
- Diagnosis: 1.478
- Age: 1.084
- Gender : 1.187
- Family: 1.160

Interpretation: All VIF values are well below 5, indicating no problematic collinearity.

**5. Model Fit – Pseudo-R² for Generalized Mixed Model (MuMIn package)**

Function used: r.squaredGLMM(mod_granMaxfun)

- Marginal R² (fixed effects only): 0.090
- Conditional R² (fixed + random effects): 0.770

_Interpretation: Fixed effects explain ~9% of the variance; ; the full model including random effects explains ~77%.

**Model **4. Mod**el Diagnostics and Assumption Checks. Lagged model with impulsivity as dependent variable.**

**1. Residual Diagnostics – DHARMa Package**

**a. Dispersion Test**
DHARMa nonparametric dispersion test via sd of residuals fitted vs. simulated
Data: simulationOutput
Dispersion = 0.927
p-value = 0.536
Alternative hypothesis: two.sided

**b. Zero-Inflation Test**
DHARMa zero-inflation test via comparison to expected zeros with simulation under H0
Data: simulationOutput
Ratio observed/expected zeros = 1.0182
p-value = 0.904
Alternative hypothesis: two.sided

Interpretation: Neither overdispersion nor zero-inflation was detected in the model residuals.

**Supplementary Figure 10. Histogram of Expected vs. Simulated Zeros (DHARMa Zero-Inflation Test)**


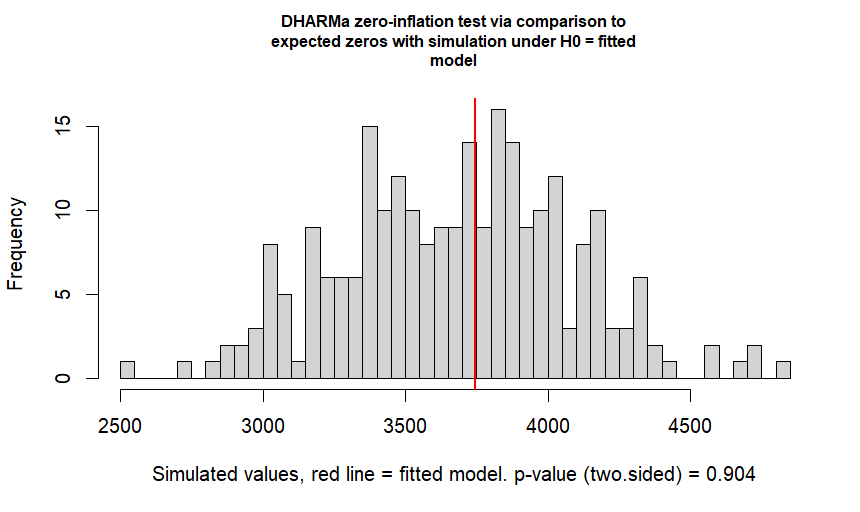


Interpretation: The distribution of simulated zeros matches the observed frequency, indicating no evidence of zero-inflation.

**2. Residuals vs. Predictor**

Plot of standardized residuals against ImpulTotal_Center (rank-transformed) to assess non-linearity or heteroscedasticity.

**Supplementary Figure 11. Residuals vs. Impulsivity Predictor (DHARMa)**


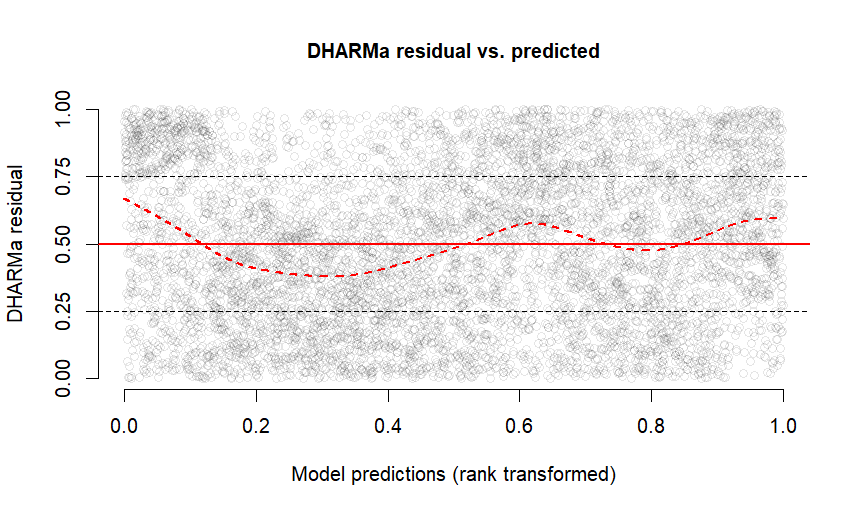
Interpretation: No clear pattern is visible in the residuals, suggesting the absence of major violations in linearity or homoscedasticity with respect to impulsivity.

**3. Normality of Random Effects**

QQ-plots of random intercepts and slopes to visually inspect normality assumptions.

**Supplementary Figure 12. QQ-plot of Random Effects**


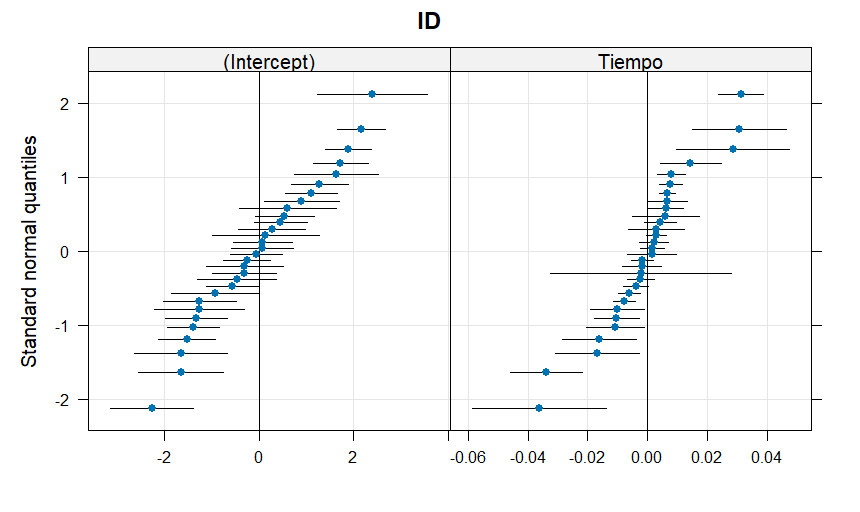
Interpretation: Both the intercept and slope random effects closely follow the expected normal distribution, supporting the assumption of normality in the random effects structure.

**4. Multicollinearity Diagnostics – VIF (Linear Approximation)**

Variance Inflation Factors (VIF) from an approximate linear model (lm):

- Time: 1.049
- Depression (Time invarying): 1.545
- Depression (Time varying): 1.001
- Diagnosis: 1.478
- Age: 1.084
- Gender : 1.187
- Family: 1.160

Interpretation: All VIF values are well below 5, indicating no problematic collinearity.

**5. Model Fit – Pseudo-R² for Generalized Mixed Model (MuMIn package)**

Function used: r.squaredGLMM(mod_granMaxfun)

- Marginal R² (fixed effects only): 0.090
- Conditional R² (fixed + random effects): 0.766

_Interpretation: Fixed effects explain ~9% of the variance; ; the full model including random effects explains ~77%.
