# Supplementary material for: The relationships between impulsivity and mood in bipolar disorder: An ecological momentary assessment study
Source: PLoS One. 2025 Jul 2;20(7):e0314963. doi: 10.1371/journal.pone.0314963 (PMC12221055; doi:10.1371/journal.pone.0314963)
Supplement: Attachment — Submitted filename: PLOS One_reviewer.docx [file pone.0314963.s002.docx]

Thank you for submitting to PLOS One. Here are some suggestions for the manuscript titled *"The relationships between impulsivity and mood in bipolar disorder: An ecological momentary assessment study."*

**Major Concerns**

1. **Sample Size & Generalizability**
   - The study includes only 30 participants across four groups, which is quite small for making strong conclusions. The authors should acknowledge this limitation more explicitly and discuss how it affects the generalizability of the findings.
   - Were any power analyses conducted to determine whether the sample size sufficiently detected significant effects?
2. **Measurement of Impulsivity and Mood**
   - The ecological momentary assessment (EMA) measure of impulsivity relies on four self-reported items that have not been fully validated. This should be more explicitly acknowledged as a limitation.
   - Mood was measured using a single-item scale (0-100). While EMA is useful for momentary assessment, using a more comprehensive mood scale (e.g., PANAS, PHQ-9) might provide a richer picture.
3. **Statistical Model Clarity**
   - The mixed-effects regression models used are appropriate, but explaining how lagged effects were handled could be clearer.
   - It would be helpful to clarify whether the results controlled for individual variability in impulsivity and mood trends over time.
4. **Direction of Causality**
   - The study finds that impulsivity predicts lower mood at the following assessment, but mood does not predict impulsivity. This contradicts some prior EMA studies (e.g., Titone et al., 2022). The authors should discuss alternative explanations, such as possible mediators (e.g., stress or sleep quality).
   - Reverse causality (mood influencing impulsivity) should be discussed more thoroughly.

**Minor Concerns**

1. **Clarify Inclusion/Exclusion Criteria**
   - The exclusion of individuals with substance use disorders is reasonable but may have removed an essential subgroup in BD. A brief discussion on how this impacts the results would be helpful.
2. **More Balanced Discussion of Findings**
   - The discussion focuses primarily on impulsivity as a risk factor for mood changes. However, prior studies suggest a bidirectional relationship. The authors should provide a more nuanced interpretation of how their findings fit into existing literature.
3. **Consistency in Terminology**
   - The term *“multiplex families”* is used throughout but is not clearly defined in the introduction. Adding a brief definition (e.g., “families with multiple first-degree relatives diagnosed with BD”) would be helpful.
4. **References and Citations**
   - Some references (e.g., Titone et al., 2022; Deep et al., 2016) are mentioned, but their findings could be contrasted more explicitly with the present results.

**Overall Recommendation**

The study is well-structured and addresses an important topic in BD research. However, given the small sample size and limitations of impulsivity measurement, the conclusions should be more cautious. A more explicit discussion of alternative explanations and prior inconsistent findings would strengthen the manuscript.
