# Supplementary material for: The relationships between impulsivity and mood in bipolar disorder: An ecological momentary assessment study
Source: PLoS One. 2025 Jul 2;20(7):e0314963. doi: 10.1371/journal.pone.0314963 (PMC12221055; doi:10.1371/journal.pone.0314963)
Supplement: Attachment — Submitted filename: Reviewer_response.docx [file pone.0314963.s004.docx]

Reviewer #1: Review Summary

This study explores the relationship between impulsivity and mood in individuals with bipolar disorder (BD) using ecological momentary assessment (EMA). By capturing real-time fluctuations in impulsivity and mood, the study provides valuable insights into their dynamic interplay. Additionally, the inclusion of multiplex family participants offers an interesting perspective on potential genetic and environmental influences. The use of mixed-effects regression models to examine both concurrent and lagged effects further strengthens the methodological approach.

Despite these strengths, several critical issues need to be addressed before the manuscript can be considered for publication.The most pressing concern is the potential overfitting of the statistical models given the small sample size (n=30).The use of complex mixed-effects regression models, particularly the lagged analyses, may not be fully appropriate for a dataset of this size and could lead to unstable or misleading results. The manuscript would also benefit from better data visualization, a more thorough discussion of the findings in relation to existing literature, and greater clarity regarding data-sharing practices.


Strengths

This study is valuable in several ways. First, it addresses an important clinical issue by examining the relationship between impulsivity and mood instability in BD, which has significant implications for treatment and symptom management. Second, the use of EMA allows for real-time data collection, reducing recall bias and improving the ecological validity of the findings. Third, the inclusion of multiplex family participants adds a novel dimension to the study, enabling an exploration of potential genetic and familial influences on impulsivity and mood regulation. Finally, the application of mixed-effects regression models is methodologically sophisticated and allows for a nuanced understanding of both concurrent and time-lagged relationships.

Areas for Improvement
1. Addressing Potential Overfitting in Statistical Models
One of the primary concerns with this study is the potential overfitting of the statistical models. The use of mixed-effects regression models, particularly with a small sample size of only 30 participants, may lead to unreliable parameter estimates and reduced generalizability. The inclusion of multiple predictors in these models, combined with the complexity of lagged analyses, increases the risk of overfitting and statistical instability.
To address this, I strongly recommend that the authors assess the risk of overfitting by examining model assumptions, variance inflation factors, and potential multicollinearity. Additionally, alternative statistical approaches should be considered to validate the robustness of the findings. For instance, conducting repeated measures ANOVA or generalized linear mixed models (GLMM) may provide a more appropriate way to analyze the data given the sample size. If the authors choose to retain the current modeling approach, they should justify why mixed-effects models are necessary and discuss the limitations of applying such complex methods with a small dataset.

Suggested Revisions:
- Assess overfitting risks and confirm model assumptions.
- Conduct additional analyses using simpler statistical models and compare results.
- Justify the use of mixed-effects models and acknowledge their limitations.

Thank you very much for your valuable suggestions. We greatly appreciate your dedication and helpful recommendations aimed at improving our analyses and enhancing the robustness of the study. The reviewer correctly identified important concerns related to the modeling approach. Upon examining model assumptions, we detected heteroscedasticity in the residuals. Consequently, we attempted transformations of the dependent variable; however, these problems persisted. To address this issue, we ultimately chose to transform the dependent variable into a dichotomous variable based on the median and performed mixed logistic regression analyses.

Additionally, we decided to remove interaction terms because adequately testing interactions would require a substantially larger sample size—far beyond our current sample—which would otherwise lead to a high risk of overfitting and unstable estimates. We have ensured that all assumptions for the revised regression models are adequately met and have provided evidence in the supplementary materials. The main findings remain consistent with those obtained from the previous models. Given that our revised logistic mixed regression models based on a dichotomized dependent variable provide stable and consistent results, we believe that additional sensitivity analyses, as proposed, would not satisfy necessary model assumptions nor provide further meaningful insights. We have updated all tables and revised the statistical analysis section accordingly.

2. Enhancing Data Visualization
The manuscript would benefit from clearer visual representation of the study’s methodology and key findings.Currently, there is no flowchart outlining participant recruitment, group classification, and study procedures,making it difficult to follow the research design. Furthermore, the results section lacks figures depicting the impulsivity-mood relationship,which would greatly aid in interpretation.
To improve clarity, I recommend including:
- A flowchart summarizing participant selection, group categorization, and data collection.
- Scatter plots with regression lines to illustrate the relationship between impulsivity and mood.
- Line graphs to show how impulsivity and mood fluctuate over time across different groups.
Suggested Revisions:
- Add a flowchart illustrating the study design.
- Include scatter plots and line graphs to enhance the presentation of results.

Thank you very much for the suggestion. The reviewer is right in pointing out that the study lacked the visual component that helps readers better understand the article and clarifies the meaning of the data. Following the reviewer’s recommendation, we have incorporated the suggested graphs as Figure 1, Figure 2, and Figure 3.

3. Strengthening the Discussion: Comparison with Existing Literature

The discussion section provides a solid interpretation of the results but could be further strengthened by placing the findings in a broader research context. Specifically, the study reports no significant difference in impulsivity between multiplex BD patients and non-multiplex BD patients,despite previous research suggesting otherwise. However, the manuscript does not sufficiently explain this discrepancy.
To enhance the discussion, I suggest exploring possible reasons why multiplex BD patients did not exhibit greater impulsivity.Factors such as sample size limitations, differences in assessment methods, or potential protective influences in multiplex families should be considered. Additionally, the study’s findings should be compared more extensively with existing research on impulsivity in BD.

Suggested Revisions:
- Discuss why the study’s findings differ from previous research.
- Consider alternative explanations (e.g., sample size, protective factors).
- Strengthen comparisons with existing literature on BD and impulsivity.

Thank you very much for this insightful comment. We agree that the discrepancy between our findings and previous research on impulsivity in multiplex BD patients required further elaboration. Following your suggestion, we have revised the discussion section to explore possible explanations, including the limited sample size, potential protective factors in multiplex families, and methodological differences in the assessment of impulsivity. We have also expanded the comparison with existing literature on impulsivity in BD, aiming to provide a more comprehensive interpretation of our results within the broader research context.

4. Clarifying Data Availability Statement

PLOS ONE encourages open data-sharing practices whenever possible. While the manuscript states that data are available upon request, providing access to the dataset through a public repository (e.g., OSF, Dryad) would be preferable. If ethical or legal constraints prevent full data sharing, a brief clarification in the manuscript would be helpful.
Suggested Revisions:
- If feasible, upload the dataset to a public repository and provide a DOI.
- If data-sharing restrictions apply, briefly explain the reason in the manuscript.

We have reconsidered the possibility of sharing the data. Our initial concern was related to the potential risk to participant confidentiality, particularly regarding the involvement of families. However, since the dataset does not contain any pedigree information and only indicates group membership at a general level, we believe the data can be shared without compromising confidentiality. Please find the link here: <https://osf.io/6qsbx/>

Thank you for submitting to PLOS One. Here are some suggestions for the manuscript titled *"The relationships between impulsivity and mood in bipolar disorder: An ecological momentary assessment study."*

**Major Concerns**

1. **Sample Size & Generalizability**
   - The study includes only 30 participants across four groups, which is quite small for making strong conclusions. The authors should acknowledge this limitation more explicitly and discuss how it affects the generalizability of the findings.
   - Were any power analyses conducted to determine whether the sample size sufficiently detected significant effects?

We appreciate the reviewer’s thoughtful comment regarding the sample size. We agree that the small number of participants (n=30), despite the intensive longitudinal design, constitutes a key limitation of the present study. We have now revised the *Limitations* section of the manuscript to more explicitly acknowledge that the limited sample may reduce statistical power, increase the risk of Type II errors, and constrain the generalizability.

Additionally, we clarify that no a priori power analysis was conducted. This study was designed as an exploratory investigation of within-person mood-impulsivity dynamics using ecological momentary assessment (EMA) in a rare and complex sample that includes participants from multiplex bipolar disorder families. While the dense momentary data provided valuable insights, we agree that future studies with larger and more diverse samples will be essential to confirm and extend our findings.

2. **Measurement of Impulsivity and Mood**
   - The ecological momentary assessment (EMA) measure of impulsivity relies on four self-reported items that have not been fully validated. This should be more explicitly acknowledged as a limitation.
   - Mood was measured using a single-item scale (0-100). While EMA is useful for momentary assessment, using a more comprehensive mood scale (e.g., PANAS, PHQ-9) might provide a richer picture.

We thank the reviewer for highlighting this important point. We agree that the EMA measure of impulsivity, although based on the Momentary Impulsivity Scale recommended for EMA contexts, consists of four self-reported items that have not been fully validated in clinical populations. We have now acknowledged this more explicitly as a limitation in the manuscript.

Similarly, we recognize that mood was assessed using a single-item visual analogue scale ranging from 0 to 100, which may not fully capture the complexity of affective states. While the use of brief measures is often necessary in EMA to minimize participant burden and maintain compliance, we acknowledge that more comprehensive scales (e.g., PANAS or PHQ-9) might provide a richer and more nuanced assessment of mood. This point has also been added to the *Limitations* section to provide a more balanced view of the methodological constraints.

3. **Statistical Model Clarity**
   - The mixed-effects regression models used are appropriate, but explaining how lagged effects were handled could be clearer.
   - It would be helpful to clarify whether the results controlled for individual variability in impulsivity and mood trends over time.

We thank the reviewer for this helpful suggestion. We have now clarified in the *Statistical analysis* section how lagged effects were modeled using prior assessments (t-1), and how time-varying predictors were decomposed to distinguish within- and between-person effects. Furthermore, we confirm that our models included random intercepts and random slopes for time at the participant level, which allowed us to control for individual differences in mood and impulsivity trajectories over time.

4. **Direction of Causality**
   - The study finds that impulsivity predicts lower mood at the following assessment, but mood does not predict impulsivity. This contradicts some prior EMA studies (e.g., Titone et al., 2022). The authors should discuss alternative explanations, such as possible mediators (e.g., stress or sleep quality).
   - Reverse causality (mood influencing impulsivity) should be discussed more thoroughly.

We appreciate the reviewer's valuable suggestions and agree that the study would benefit from further discussion on this aspect.

In the new version of the manuscript, we have incorporated a more detailed explanation of the discrepancies between our findings and those of previous studies, considering possible factors that might have contributed to these mixed results, such methodological differences, participants' clinical status, and small sample size.

**Minor Concerns**

1. **Clarify Inclusion/Exclusion Criteria**
   - The exclusion of individuals with substance use disorders is reasonable but may have removed an essential subgroup in BD. A brief discussion on how this impacts the results would be helpful.

Thank you for this observation. We have added a brief note in the *Limitations* section acknowledging that excluding individuals with substance use disorders may limit the generalizability of the findings, as this subgroup is often present in BD and may present distinct clinical features.

2. **More Balanced Discussion of Findings**
   - The discussion focuses primarily on impulsivity as a risk factor for mood changes. However, prior studies suggest a bidirectional relationship. The authors should provide a more nuanced interpretation of how their findings fit into existing literature.

We thank the reviewer for this observation.

In the revised version of the manuscript, we have expanded the discussion to contextualise our results within the existing body of literature that has found bidirectional relationships between impulsivity and mood. Furthermore, we have emphasised that, although our results show a unidirectional association from impulsivity to mood, this interpretation should be considered preliminary and subject to verification in future research with larger samples and diverse methodological designs.

3. **Consistency in Terminology**
   - The term *“multiplex families”* is used throughout but is not clearly defined in the introduction. Adding a brief definition (e.g., “families with multiple first-degree relatives diagnosed with BD”) would be helpful.

We thank the reviewer for this helpful comment. We have now added a brief definition of "multiplex families" in the Introduction, clarifying that these are families with a high prevalence of bipolar disorder and an increased burden of genetic risk variants. We have also referenced prior work from our group using this type of sample to provide further context.

4. **References and Citations**
   - Some references (e.g., Titone et al., 2022; Deep et al., 2016) are mentioned, but their findings could be contrasted more explicitly with the present results.

We thank the reviewer for this valuable observation. We have revised and expanded the discussion section to more explicitly contrast the findings of previous studies, such as those of Titone et al. (2022) and Depp et al. (2016), with the results obtained in the present study.

This more detailed comparison allows us to place our findings within the framework of the existing literature and highlights the need for further research on the relationship between impulsivity and mood in diverse clinical samples.

**Overall Recommendation**

The study is well-structured and addresses an important topic in BD research. However, given the small sample size and limitations of impulsivity measurement, the conclusions should be more cautious. A more explicit discussion of alternative explanations and prior inconsistent findings would strengthen the manuscript.
